# Non-operative management of pneumoperitoneum following cardiopulmonary resuscitation

**DOI:** 10.1093/jscr/rjac219

**Published:** 2022-05-18

**Authors:** Christopher L Johnson, Camilla Gomes, Justin Cheng, Carter C Lebares

**Affiliations:** School of Medicine, University of California San Francisco, San Francisco, CA 94143, USA; Department of Surgery, University of California San Francisco, San Francisco, CA 94143, USA; Department of Surgery, Division of Plastic and Reconstructive Surgery, University of California San Francisco, San Francisco, CA 94143, USA; School of Medicine, University of California San Francisco, San Francisco, CA 94143, USA

## Abstract

Spontaneous pneumoperitoneum in a patient with a tracheostomy tube following cardiopulmonary resuscitation is exceedingly rare, with little experimental nor observational data to guide evidence-based management. We present the case of a 75-year-old woman with a tracheostomy tube who developed pneumoperitoneum following CPR. The patient experienced pulseless electrical activity arrest requiring nine rounds of chest compressions to return to spontaneous circulation. Computerized tomography demonstrated pneumothoraces, subcutaneous emphysema and extensive intraperitoneal, extraperitoneal and retroperitoneal free air without evidence of visceral perforation. The patient’s abdomen was distended without tenderness, guarding or rebound. She had a white blood cell count mildly elevated from her baseline levels. The management plan of serial abdominal exams without operative intervention was chosen given the absence of clinical and laboratory signs of peritonitis. This case highlights the importance of developing a standardized management algorithm for patients with pneumoperitoneum in the setting of tracheostomy tubes without evidence of perforation.

## INTRODUCTION

Pneumoperitoneum is most commonly associated with gastric and duodenal ulcer, perforated appendix, perforated colon diverticulum and positive pressure ventilation [1, 2]. These patients frequently present with acute abdominal pain, signs of peritonitis, fever, leukocytosis and management includes emergent surgical intervention [3, 4]. Following CPR, pneumoperitoneum frequently indicates gastrointestinal perforation secondary to thoracic and abdominal visceral tears occurring during airway management, rescue breathing and compressions [3–6]. Here, we present the case of a patient with a tracheostomy tube who developed pneumoperitoneum following CPR.

## CASE REPORT

A 75-year-old woman was admitted to a secondary community hospital for shortness of breath and intermittent chest tightness. Further evaluation at the hospital revealed diffuse ST-elevation myocardial infarction. She underwent placement of an intra-aortic balloon pump, multiple coronary artery angiographies and coronary artery stent placements. Despite these therapies, the patient continued to have worsening cardiogenic shock. On hospital Day 2, the patient was transferred to a tertiary care facility for higher-level management. On presentation, she was found to have multifactorial shock, with broad-spectrum antibiotics added for the septic component.

Twenty days after transfer, the patient was in the transition care unit when she experienced pulseless electrical activity (PEA) arrest, believed to be secondary to hypoxia in the setting of bleeding tracheostomy and airway obstruction due to clot. She underwent nine rounds of chest compressions with concurrent ventilation via the tracheostomy tube, with successful return of spontaneous circulation. At the end of the code, patient noted to have one round of melanotic stool, an enlarging left groin hematoma, abdominal distension, and a decrease in hemoglobin to 7.0 g/dl down from 9.3 g/dl. Due to concerns of possible bleeding, computerized tomography (CT) scan of her chest through pelvis was ordered. Patient was transferred to the intensive care unit.

**Figure 1:**
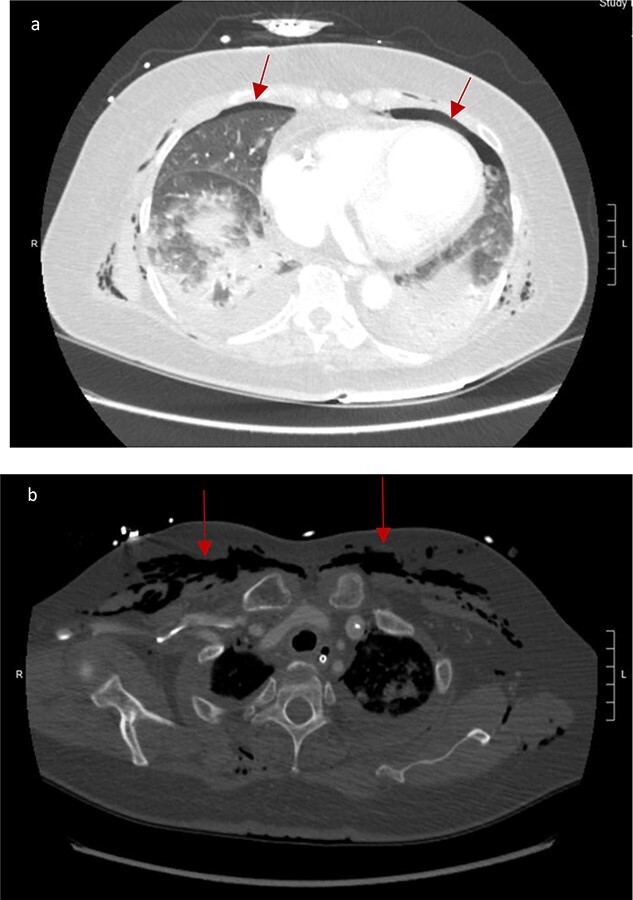
(**a**) Bilateral anterior pneumothoraces; (**b**) moderate volume of subcutaneous emphysema in the anterior chest wall.

**Figure 2:**
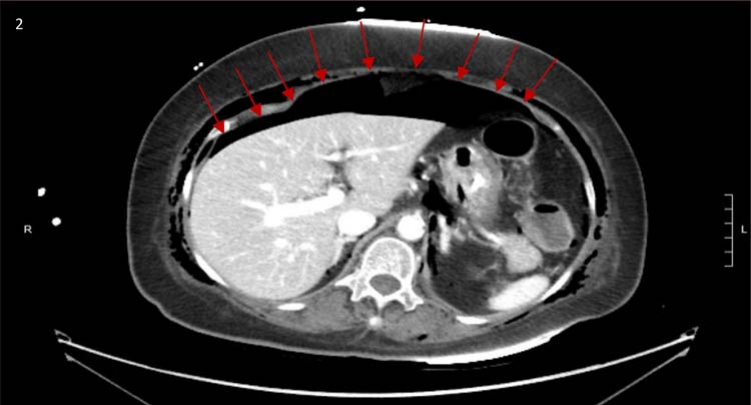
Large pneumoperitoneum with subcutaneous emphysema.

**Figure 3:**
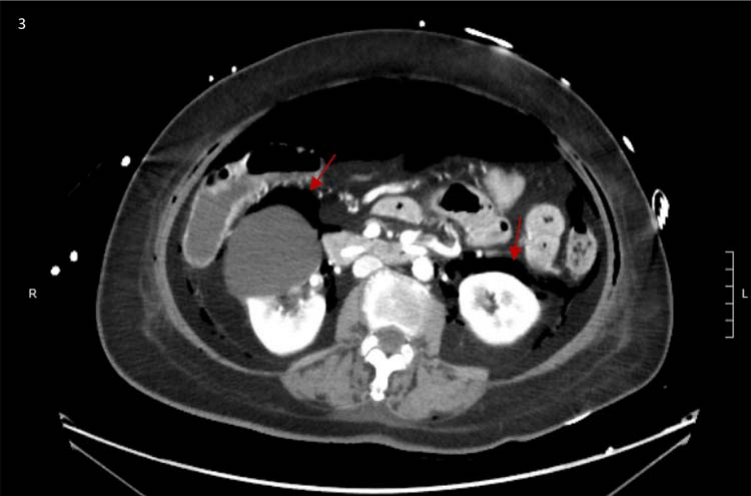
Bilateral retroperitoneal air surrounding right and left kidneys.

CT scan showed small bilateral pneumothoraces (Fig. 1a) in the setting of several mildly displaced anterior rib fractures with associated subcutaneous emphysema (Fig. 1b), as well as extensive intraperitoneal (Fig. 2), extraperitoneal and retroperitoneal (Fig. 3) air. However, imaging was negative for evidence of perforation. Given concern for abdominal compartment syndrome as evidenced by abdominal distension on exam, initially elevated peak pressures to 24 immediately following intubation, and CT findings of pneumoperitonum, the General Surgery service was consulted for evaluation and further assistance with management. On clinical exam, the patient’s abdomen was distended without tenderness, guarding, or rebound. Laboratory results were notable for white blood cell count of 21.1 per mm^3^, platelet count of 314 per mm^3^, and a whole blood lactate of 8.1 mmol/l. Her leukocytosis, though only mildly increased from her baseline of 15 over the previous days, was attributed to known Staphylococcus bacteremia for which she was on antibiotic therapy with a contribution from her shock and PEA arrest. Given the patient was hemodynamically stable with no signs of an acute abdomen, we decided to manage with serial abdominal exams without operative intervention. The patient was closely followed over the subsequent 5 days, during which time she remained stable; she no longer required pressors and was weaned to trach collar on post-arrest Day 2; her white blood cell count continued to downtrend and normalized post-arrest Day 7 as she remained on antibiotic therapy; no additional abdominal imaging was obtained, given her very benign abdominal exam. Operative management was never utilized and she remained asymptomatic. While in the intensive care unit, the patient improved and she was able to engage in physical and occupational therapy despite the pneumothoraces. She was discharged to a long-term acute care hospital on hospital Day 36.

## DISCUSSION

The presence of abdominal free air in the absence of clinical and laboratory signs of peritonitis has been termed ‘nonsurgical’, ‘spontaneous’ and ‘idiopathic’ pneumoperitoneum. CPR has been documented to cause benign pneumoperitoneum, without evidence of gastrointestinal perforation [1, 7]. While numerous articles propose a workup algorithm for patients with hemodynamically stable pneumoperitoneum that utilize serial abdominal exam and imaging studies to assess for evolving indications for emergent surgical intervention, there is little written that focuses specifically on CPR in the setting of tracheostomy tube [2, 7–9]. Abdominal and thoracic etiologies of spontaneous pneumoperitoneum include abdominal surgical laparoscopy, chronic abdominal peritoneal dialysis, percutaneous endoscopic gastrostomy tube placement, diagnostic colonoscopy, spontaneous peritonitis, trauma and cardiac transplant [10].

The extent of subcutaneous and intraperitoneal air found in this patient without evidence to support a perforation etiology posed a diagnostic challenge. We propose that the physiologic disruption of tissue planes that occur during tracheostomy creation were aggravated during compressions and positive ventilation, allowing air to track into the subcutaneous tissue. While it is difficult to specify how the air settled intra-abdominally, it is possible that her rib fractures punctured her peritoneum, allowing the subcutaneous air to track into the abdomen.

CPR is generally associated with small pneumothoraces; however, the presence of the tracheostomy tube allowed massive pneumoperitoneum to develop post-CPR. Our patient presented with imaging evidence of extensive pneumoperitoneum without signs and clinical evaluation demonstrated that operative intervention was not necessary despite the extensive pneumoperitoneum. Watchful waiting can be employed in the setting of a benign abdominal exam and stable laboratory values with a plan for surgical intervention should evidence of hemodynamic instability immerge.
